# Circulator function in a Josephson junction circuit and braiding of Majorana zero modes

**DOI:** 10.1038/s41598-021-81503-1

**Published:** 2021-01-19

**Authors:** Mun Dae Kim

**Affiliations:** College of Liberal Arts, Hongik University, Sejong, 30016 Korea

**Keywords:** Quantum information, Qubits, Superconducting devices

## Abstract

We propose a scheme for the circulator function in a superconducting circuit consisting of a three-Josephson junction loop and a trijunction. In this study we obtain the exact Lagrangian of the system by deriving the effective potential from the fundamental boundary conditions. We subsequently show that we can selectively choose the direction of current flowing through the branches connected at the trijunction, which performs a circulator function. Further, we use this circulator function for a non-Abelian braiding of Majorana zero modes (MZMs). In the branches of the system we introduce pairs of MZMs which interact with each other through the phases of trijunction. The circulator function determines the phases of the trijunction and thus the coupling between the MZMs to gives rise to the braiding operation. We modify the system so that MZMs might be coupled to the external ones to perform qubit operations in a scalable design.

While the ultimate goal of practical quantum computer is still far away, the noisy intermediate-scale quantum (NISQ) computing^1^ is expected to be realized in the near future due to the remarkable advancement in the qubit coherence and control. The quantum supremacy that quantum device can solve a problem that no classical computer can solve in any feasible amount of time is regarded as a notable milestone^2^. The programmable NISQ computing for quantum supremacy requires a scalable design of quantum circuit, which is severely challenging. We, here, provide an approach to cope with this challenge by proposing a scheme for a circulator function which enables selective coupling between arbitrary two branches at a trijunction by using a three-Josephson junction flux qubit as a control element in a superconducting circuit^3−6^.

In this study we introduce a three-Josephson junction loop consisting of three small loops with three branches and a trijunction as shown in Fig. 1a. Usually the Hamiltonians of the superconducting circuit with threading fluxes for quantum information processing have been provided phenomenologically. The effective potential in the Hamiltonian is given in an approximate way so that the form and the coefficients have not been precisely derived from the first principle. For the understanding of the system we need to know the exact form of the Hamiltonian and the process by which the Hamiltonian is obtained. For the superconducting loop system in the present study we derive the Lagrangian of system exactly from fundamental boundary conditions and obtain the effective potential of the system analytically. This Lagrangian describes the circulating function in the ground state of the system, where we can selectively couple two branches to flow currents while the other branch does not. This kind of study will help analyzing other systems for quantum information processing.

Circulator is a nonreciprocal three-port device that routes a signal to the next port. For the universal quantum computing quantum gates between different two qubits in a scalable design is required. Hence the circulator function which enables selective coupling between arbitrary two qubits among several qubits has been studied intensively. Recently Josephson junction based on-chip circulators much smaller than commercial microwave circulator have been proposed for the quantum information processing with superconducting devices^5,7^. The superconductor-based circulators have remarkably small photon losses compared to the commercial nonreciprocal ferrite circulators^8^. Moreover, the superconductor-based circulators are much smaller than the commercial circulators so that they can be integrated into a scalable circuit.

By piercing a magnetic flux into one of three small loops we are able to make the current flow between two branches selectively in situ, while the other is isolated, resulting in the circulator function. Usually the circulator routes a signal from one port to the other. Present design, in contrast, performs a circulator function that routes a signal between two branches at a trijunction in a closed circuit rather than transferring the signal to outer port. In this way, we can connect arbitrary pair of branches to perform quantum gate operations. For the NISQ computing we need to perform the circulator function in a scalable circuit where the trijunctions are connected with each other to form a lattice structure. We thus consider an improved design where the trijunction is located outside of the loop as shown in Fig. 1b, which is topologically equivalent with the design in Fig. 1a.

Further, we can use the circulator function to realize the braiding of Majorana zero modes (MZM)^9,10^ for topological quantum computing^11,12^. Topologically-protected quantum processing is expected to provide a path towards fault-tolerant quantum computing. Since quantum states are susceptible to environmental decoherence, protection from local perturbation is an emergent challenge for quantum information processing. Non-Abelian states are the building block of topological quantum computing carrying the nonlocal information. The nonlocally encoded quantum information is resilient to local noises and, if the temperature is smaller than the excitation gap, temporal excitation rate is exponentially suppressed. Majorana zero modes, $\gamma$, are predicted to exhibit non-Abelian exchange statistics, and they are self-adjoint $\gamma ^\dagger =\gamma$ in contrast to ordinary fermion operators. The theoretically proposed structures attracted a great deal of intention to realizing MZMs in condensed matter systems. MZMs are predicted to emerge in $\nu =5/2$ fractional quantum Hall states^11,13^, p-wave superconductors^14,15^, and one- or two-dimensional semiconductor/superconductor hybrid structures^16^. The branches in our scheme for braiding contains semiconductor/superconductor hybrid structures with p-wave-like superconductivity induced from s-wave superconductors via proximity effect.

In two-dimensional spinless $p+ip$ topological superconductors MZMs are hosted in vortices or in the chiral edge modes as localized Andreev-bound zero-energy states at the Fermi energy. The p-wave-like superconductivity can be induced from s-wave superconductors via proximity effect in a hybrid structure^17^. Semiconductor thin film with Zeeman splitting and proximity-induced s-wave superconductivity has been expected to be a suitable platform for hosting MZMs^18^. On the other hand, the one-dimensional semiconducting nanowire has also been shown to provide MZMs at the ends of the nanowire^19^. The MZMs should be prepared, braided, and fused to implement qubit operations. In one-dimensional wire the braiding is not well defined, which can be overcome in a wire network of trijunction. However, the original scheme^17^ with Josephson trijunction has not yet been explored.

Recently, an experimental evidence of MZM in a trijunction has been reported^20^. The nanowire trijunctions are manipulated by the chemical potential^21^, the charging energy^22^, and the phase^23^. In the present study a pair of MZMs can be introduced in each branch near the trijunction of Fig. 1b. Three MZMs of each pair are coupled through Josephson junctions with phase differences $\varphi '_1,\varphi '_2,$ and $\varphi '_3$ in the system. The three Josephson junction loop controls the selective coupling among three MZM pairs. By applying a threading flux into one of the loops of Fig. 1b we can use the circulator function to control the phases $\phi '_i$ and thus the couplings among MZMs in the trijunction to perform the braiding operation and, further, quantum gate operations. In contrast to the previous phase modulation scheme^23^ trying to switch off the current mediated by MZMs which are inside of the loop the present proposal uses circulating function to perform braiding operations. Further, our scheme enables the interaction between MZMs outside so that we may provide a scalable design in a one or two-dimensional lattice system for coupling between MZMs which belong to different trijunctions.

## Results

### Three-Josephson junction loop with a trijunction

The precise fluxoid quantization condition of superconducting loop reads $-\Phi _t+(m_c/q_c)\oint {\vec v}_c\cdot d{\vec l}=n\Phi _0$ with ${\vec v}_c$ being the average velocity of Cooper pairs, $q_c=2e$ the Cooper pair charge, and $m_c=2m_e$ the Cooper pair mass^24,25^. The total magnetic flux $\Phi _t$ threading the loop is the sum of the external and the induced flux $\Phi _t=\Phi _\text{ext}+\Phi _{\mathrm{ind}}$. With the superconducting unit flux quantum $\Phi _0=h/2e$ we introduce the reduced fluxes, $f_t=\Phi _t/\Phi _0=f+f_{\mathrm{ind}}$ with $f=\Phi _{\mathrm{ext}}/\Phi _0$ and $f_{\mathrm{ind}}=\Phi _{\mathrm{ind}}/\Phi _0$, expressing the fluxoid quantization condition as $kl=2\pi (n+f_t)$ with *l* being the circumference of the loop, *k* the wave vector of the Cooper pair wavefunction and *n* an integer.

**Figure 1:**
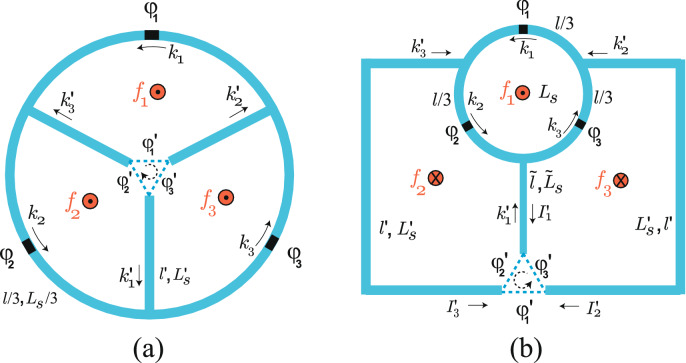
(**a**) Three-Josephson junction loop with length *l* and geometric inductance $L_s$ has three Josephson junctions with phase differences $\varphi _i$ and three branches with length $l'$ and geometric inductance $L'_s$. $k_i$ and $k'_i$ are the wave vectors of the Cooper pairs and $f_i$ the external flux threading the loops. $\varphi '_i$’s are the trijunction phase differences. (**b**) A scheme that three branches and trijunction are extracted out from the three-Josephson junction loop and turned over: left and right branches have length $l'$ and geometric inductance $L'_s$ and central branch ${{{\tilde{l}}}}$ and ${{{\tilde{L}}}}_s$. Two schemes in (**a**,**b**) are topologically equivalent with each other.

The scheme in Fig. 1a consists of three-Josephson junction loop and three small loops with threading fluxes $f_i=\Phi _{\mathrm{ext,i}}/\Phi _0$. The fluxoid quantization conditions around three loops, including the phase differences $\varphi _i$ and $\varphi '_i$ across the Josephson junctions, are represented as the following periodic boundary conditions^26,27^,

1 $$\begin{aligned} k_1\frac{l}{3}-k'_3l'+k'_2 l'+\varphi _1+\varphi '_1= & {} 2\pi (m_1+f_1+f_{\mathrm{ind,1}}), \end{aligned}$$

2 $$\begin{aligned} k_2\frac{l}{3}-k'_1l'+k'_3 l'+\varphi _2+\varphi '_2= & {} 2\pi (m_2+f_2+f_{\mathrm{ind,2}}),\end{aligned}$$

3 $$\begin{aligned} k_3\frac{l}{3}-k'_2 l'+k'_1l'+\varphi _3+\varphi '_3= & {} 2\pi (m_3+f_3+f_{\mathrm{ind,3}}), \end{aligned}$$

where $k_i, l$, and $l'$ are the wave vector of Cooper pairs, the length of the three-Josephson junction loop, and three branches, respectively, and $m_i$’s are integer. Here, $\varphi _i$’s are the phase differences of Josephson junctions in the three-Josephson junction loop and $\varphi '_i$’s phase differences of the trijunction whose positive direction, we choose, is clockwise as shown in Fig. 1a. Which branches carry current, while the other not, is determined by threading a flux, $f_i$, into a specific loop.

The induced flux $f_{\mathrm{ind,1}}$, for example, can be written as $f_{\mathrm{ind,1}}=\Phi _{\mathrm{ind,1}}/\Phi _0=(1/\Phi _0)(L_sI_1/3+L'_sI'_2-L'_sI'_3)$, where the Cooper pair current $I_i$ is given by

4 $$\begin{aligned} I_i=-(n_cAq_c/m_c)\hbar k_i \end{aligned}$$

with the Cooper pair density $n_c$ and the cross section *A* of the loop. The induced flux, $\Phi _{\mathrm{ind,1}}$, consists of contributions from three conducting lines, $L'_sI'_3, L'_sI'_2$ and $L_sI_1/3$, where $L_s$ and $L'_s$ are the geometric inductance of the three-Josephson junction loop and a branch, respectively, and the inductance of one third of the loop contributes to the induced flux. Further, we introduce the kinetic inductances $L_K=m_cl/An_cq^2_c$ and $L'_K=m_cl'/An_cq^2_c$^25,28,29^, and then the induced fluxes become $f_{\mathrm{ind,1}}=-(1/2\pi )[(L'_s/L'_K)(k'_2-k'_3)l'+(L_s/L_K)k_1l/3]$, $f_{\mathrm{ind,2}}=-(1/2\pi )[(L'_s/L'_K)(k'_3-k'_1)l'+(L_s/L_K)k_2l/3]$, and $f_{\mathrm{ind,3}}=-(1/2\pi )[(L'_s/L'_K)(k'_1-k'_2)l'+(L_s/L_K)k_3l/3]$ to represent the boundary conditions as

5 $$\begin{aligned} \left( 1+\frac{L_s}{L_K}\right) k_1\frac{l}{3} +\left( 1+\frac{L'_s}{L'_K}\right) (k'_2-k'_3)l'= & {} 2\pi \left( m_1+f_1-\frac{\varphi _1+\varphi '_1}{2\pi }\right) \end{aligned}$$

6 $$\begin{aligned} \left( 1+\frac{L_s}{L_K}\right) k_2\frac{l}{3} +\left( 1+\frac{L'_s}{L'_K}\right) (k'_3-k'_1)l'= & {} 2\pi \left( m_2+f_2-\frac{\varphi _2+\varphi '_2}{2\pi }\right) \end{aligned}$$

7 $$\begin{aligned} \left( 1+\frac{L_s}{L_K}\right) k_3\frac{l}{3}+\left( 1+\frac{L'_s}{L'_K}\right) (k'_1-k'_2)l'= & {} 2\pi \left( m_3+f_3-\frac{\varphi _3+\varphi '_3}{2\pi }\right) . \end{aligned}$$

In the system of Fig. 1a three Josephson junctions with $\varphi '_i$ compose a trijunction which satisfies the periodic boundary condition $\varphi '_1+\varphi '_2+\varphi '_3=2\pi n'$ with an integer $n'$. By using this condition and summing above three equations we can check that the boundary condition for three-Josephson junction loop can be expressed as $\left( 1+L_s/L_K\right) (k_1+k_2+k_3)(l/3)=2\pi \left[ n+f_1+f_2+f_3-(\varphi _1+\varphi _2+\varphi _3)/2\pi \right]$ with an integer *n*, which can also be derived directly from the fluxoid quantization condition. If we assume the superconducting branches in Fig. 1a have the same cross section *A* and Cooper pair density $n_c$ in Eq. (4), the current conservation conditions, $I_1=I_3+I'_2, I_2=I_1+I'_3$, and $I_3=I_2+I'_1$, at the nodes of three-Josephson junction loop give rise to the relations,

8 $$\begin{aligned} k_1=k_3+k'_2, ~~k_2=k_1+k'_3, ~~k_3=k_2+k'_1. \end{aligned}$$

From the boundary conditions in Eqs. (5)–(7) in conjunction with the relations in Eq. (8) we can readily obtain $k_i$ and $k'_i$ in terms of $\varphi _i$ and $\varphi '_i$ as

9 $$\begin{aligned} k_i= & {} \frac{2\pi }{l}\frac{3L_K}{L'_{\mathrm{eff}}}\left( m_i+f_i -\frac{\varphi _i+\varphi '_i}{2\pi }\right) +\frac{2\pi }{l}\left( \frac{L_K}{L_{\mathrm{eff}}}-\frac{L_K}{L'_{\mathrm{eff}}} \right) \left( n+f_1+f_2+f_3-\frac{\varphi _1+\varphi _2+\varphi _3}{2\pi }\right) , \end{aligned}$$

10 $$\begin{aligned} k'_i= & {} \frac{2\pi }{l}\frac{3L_K}{L'_{\mathrm{eff}}}\left( m_{i+2}-m_{i+1} +f_{i+2}-f_{i+1}-\frac{\varphi _{i+2}+\varphi '_{i+2}}{2\pi } +\frac{\varphi _{i+1}+\varphi '_{i+1}}{2\pi }\right) , \end{aligned}$$

where the effective inductances are defined as $L_{\mathrm{eff}}\equiv L_K+L_s$ and $L'_{\mathrm{eff}}\equiv L_K+L_s+9(L'_K+L'_s)$. Here and after, the indices, *i*, are modulo 3, for example, $i+1=i+1~ \mathrm{mod} ~3$.

The dynamics of Josephson junction is described by the capacitively-shunted model, where the current relation is given by $I=-I_c\sin \phi +C{\dot{V}}=-I_c\sin \phi -C(\Phi _0/2\pi ){\ddot{\phi }}$ with the critical current $I_c$, the capacitance *C* of Josephson junction, and the voltage-phase relation, $V=-(\Phi _0/2\pi ){{{\dot{\phi }}}}$. The quantum Kirchhoff relation then becomes $-(\Phi ^2_0/2\pi L_K)(l/2\pi )k_i=-E_{J}\sin \phi _i-C(\Phi _0/2\pi )^2{\ddot{\phi }}_i$ with the Josephson coupling energy $E_J=\Phi _0I_c/2\pi$ and the current $I=-(n_cAq_c/m_c)\hbar k$. From the Lagrangian ${{{\mathscr {L}}}}=\sum _i(1/2)C_i(\Phi _0/2\pi )^2{{{\dot{\phi }}}}^2_i-U_{\mathrm{eff}}(\{\phi _i\})$ with the effective potential of the system, $U_{\mathrm{eff}}(\{\phi _i\})$, the equation of motion, $C_i(\Phi _0/2\pi )^2{\ddot{\phi }}_i=-\partial U_{\mathrm{eff}}/\partial \phi _i$, can be derived from the Lagrange equation $(d/dt)\partial {{{\mathscr {L}}}}/\partial {{{\dot{\phi }}}}_i-\partial {{{\mathscr {L}}}}/\partial \phi _i=0$. By using the quantum Kirchhoff relation the equation of motion, then, can be represented as

11 $$\begin{aligned} \frac{\Phi ^2_0}{2\pi L_K}\frac{l}{2\pi }k_i-E_{J}\sin \phi _i= -\frac{\partial U_{\mathrm{eff}}}{\partial \phi _i}. \end{aligned}$$

We can construct the effective potential $U_{\mathrm{eff}}(\{\varphi _i,\varphi '_i\})$ as follows,

12 $$\begin{aligned} U_{\mathrm{eff}}(\{\varphi _i,\varphi '_i\})= & {} \frac{3\Phi ^2_0}{2L'_{\mathrm{eff}}} \left[ \left( m_1+f_1-\frac{\varphi _1+\varphi '_1}{2\pi }\right) ^2+ \left( m_2+f_2-\frac{\varphi _2+\varphi '_2}{2\pi }\right) ^2+\left( m_3+f_3 -\frac{\varphi _3+\varphi '_3}{2\pi }\right) ^2 \right] \nonumber \\&+\left( \frac{\Phi ^2_0}{2L_{\mathrm{eff}}}-\frac{\Phi ^2_0}{2L'_{\mathrm{eff}}} \right) \left( n+f_1+f_2+f_3-\frac{\varphi _1+\varphi _2+\varphi _3}{2\pi } \right) ^2-\sum ^3_{i=1}(E_{J}\cos \varphi _i+E'_{J}\cos \varphi '_i), \end{aligned}$$

which consists of the inductive energies of the loops and Josephson junction energies with $E'_J$ being the Josephson junction energy of trijunction. We can easily check that the effective potential $U_{\mathrm{eff}}(\{\varphi _i,\varphi '_i\})$ satisfy the equation of motion in Eq. (11) for $\phi _i=\varphi _i$ with $k_i$’s in Eq. (9). The kinetic inductance $L_K$ is much smaller than the geometric inductance $L_s$. For the usual parameter regime for three-Josephson junction qubit $L_K/L_s\sim O(10^{-3})$^30^ so that we can approximate the effective inductances as $L_{\mathrm{eff}}\approx L_s$ and $L'_{\mathrm{eff}}\approx L_s+9L'_s$.

Further, the effective potential $U_\text{eff}(\{\varphi _i,\varphi '_i\})$ should also satisfy the quantum Kirchhoff relation for the phase variables $\varphi '_i$. In Fig. 1a we consider the currents ${{{\tilde{I}}}}_i$ across the Josephson junction with phases $\varphi '_i$ and $I'_i$ flowing in the branch, where the direction of ${\tilde{I}}_i$ is counterclockwise and $I'_i$ is opposite to $k'_i$ (See Fig. S1a in the Supplementary Information). Then with the current conservation relation at nodes, $I'_i={{{\tilde{I}}}}_{i+2}-{{{\tilde{I}}}}_{i+1}$, and the current relation of Josephson junction, ${\tilde{I}}_i=-I'_c\sin \varphi '_i-C'(\Phi _0/2\pi ){\ddot{\varphi }'_i}$, we have $I'_i=-(I'_c\sin \varphi '_{i+2}+C'\frac{\Phi _0}{2\pi }{\ddot{\varphi }'_{i+2}}) +(I'_c\sin \varphi '_{i+1}+C'\frac{\Phi _0}{2\pi }{\ddot{\varphi }'_{i+1}})$. Using the equation of motion, $C'_i(\Phi _0/2\pi )^2{\ddot{\varphi }}'_i=-\partial U_\text{eff}/\partial \varphi '_i$, obtained from the Lagrange equation, the quantum Kirchhoff relation reads

13 $$\begin{aligned} -\frac{\Phi ^2_0}{2\pi L_K}\frac{l}{2\pi }k'_i =\frac{\partial U_{\mathrm{eff}}}{\partial \varphi '_{i+2}}- \frac{\partial U_{\mathrm{eff}}}{\partial \varphi '_{i+1}} -E'_{J}\sin \varphi '_{i+2}+E'_{J}\sin \varphi '_{i+1}. \end{aligned}$$

We can confirm that the effective potential $U_{\mathrm{eff}}(\{\varphi _i,\varphi '_i\})$ in Eq. (12) also satisfies the quantum Kirchhoff relation in Eq. (13) with $k'_i$ in Eq. (10).

#### Limiting case

In the system of Fig. 1a we can consider the limit that the length of branches goes to zero, $l'\rightarrow 0$, and thus two nodes at the either ends of a branch collapse to a point. As a result, we have three loops with geometric inductance $L_s/3$ which meet at the trijunction. In this limit $L'_s \rightarrow 0$ and $L'_{\mathrm{eff}}\approx L_s+9L'_s \rightarrow L_{\mathrm{eff}}\approx L_s$. Hence the effective potential $U_{\mathrm{eff}}(\{\varphi _i,\varphi '_i\})$ in Eq. (12) becomes

14 $$\begin{aligned} U_{\mathrm{eff}}(\{\varphi _i,\varphi '_i\})= & {} \frac{\Phi ^2_0}{2(L_s/3)} \left[ \left( m_1+f_1-\frac{\varphi _1+\varphi '_1}{2\pi }\right) ^2+ \left( m_2+f_2-\frac{\varphi _2+\varphi '_2}{2\pi }\right) ^2+\left( m_3 +f_3-\frac{\varphi _3+\varphi '_3}{2\pi }\right) ^2 \right] \nonumber \\&-\sum ^3_{i=1}(E_{J}\cos \varphi _i+E'_{J}\cos \varphi '_i), \end{aligned}$$

which describes the inductive energies of three loops with geometric inductance $L_s/3$ and the Josephson junction energies^25,31,32^, complying with the intuitive picture.

### Circulator function

In order to perform the NISQ computing we need to construct a scalable design with the circulator function, where the trijunctions are connected to others and the current directions can be controlled *in situ* in the circuit. However, in the design in Fig. 1a the trijunction is inside of the loop so it is not possible to couple the branches with others outside. Hence we consider an improved design where the trijunction is located outside of the loop as shown in Fig. 1b. In the Supplementary Information we show an archetype for a scalable design. Actually the inner branches and the trijunction are turned over, but the design is topologically equivalent with the design in Fig. 1a. Here the length ${\tilde{l}}$ of central branch is not equal with others anymore.

**Figure 2:**
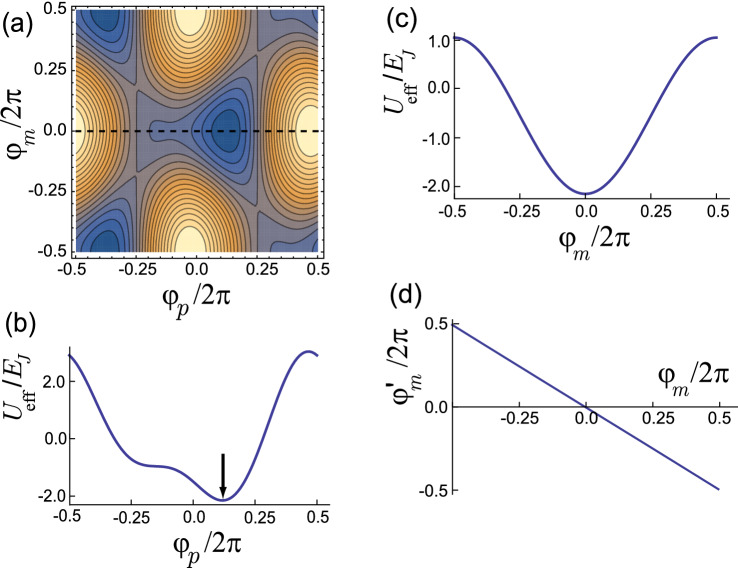
(**a**) Contour plot for the effective potential $U_{\mathrm{eff}}$ for the system in Fig. 1b as a function of $\varphi _p$ and $\varphi _m$ for $f_1=f=0.42,$ and $f_2=f_3=0$. (**b**) Profile of $U_{\mathrm{eff}}$ along the dotted line in (**a**) for $\varphi _m=0$. At $\varphi _p/2\pi \approx 0.124$ $U_{\mathrm{eff}}$ has the minimum. (**c**) The profile of $U_{\mathrm{eff}}$ for $\varphi _p/2\pi \approx 0.124$ shows $\varphi _m=0$ at the minimum of $U_{\mathrm{eff}}$ . (**d**) Plot of $\varphi '_m$ as a function of $\varphi _m$ which shows $\varphi '_m=0$ at the minimum of $U_{\mathrm{eff}}$ for $\varphi _p/2\pi \approx 0.124$ and $\varphi _m=0$.

We then introduce more general boundary conditions for the scheme in Fig. 1b including the phase differences across the Josephson junctions as

15 $$\begin{aligned} k'_2 l'-k'_3l'+k_1\frac{l}{3}+\varphi _1+\varphi '_1= & {} 2\pi (m_1+f_1-f_2-f_3+f_{\mathrm{ind,1}}), \end{aligned}$$

16 $$\begin{aligned} -k'_3 l'+k'_1{\tilde{l}}-k_2\frac{l}{3}-\varphi _2-\varphi '_2= & {} 2\pi (-m_2-f_2+f_{\mathrm{ind,2}}),\end{aligned}$$

17 $$\begin{aligned} k'_2 l'-k'_1{\tilde{l}}-k_3\frac{l}{3}-\varphi _3-\varphi '_3= & {} 2\pi (-m_3-f_3+f_{\mathrm{ind,3}}), \end{aligned}$$

with integers $m_i$. The boundary condition in Eq. (15) describes the outmost loop containing the Josephson junctions with phase differences $\varphi _1$ and $\varphi '_1$ and the conditions in Eqs. (16) and (17) the left and right loop in Fig. 1b. With the geometric and kinetic inductances ${{{\tilde{L}}}}_s$ and ${{{\tilde{L}}}}_K=m_c{\tilde{l}}/An_cq^2_c$ for the central branch, respectively, the induced fluxes become $f_{\mathrm{ind,1}}=-(1/2\pi )[(L'_s/L'_K)(k'_2-k'_3)l'+(L_s/L_K)k_1l/3], f_{\mathrm{ind,2}}=-(1/2\pi )[-(L'_s/L'_K)k'_3l'+({{{\tilde{L}}}}_s/{{{\tilde{L}}}}_K)k'_1{{{\tilde{l}}}}-(L_s/L_K)k_2l/3]$ and $f_{\mathrm{ind,3}}=-(1/2\pi )[(L'_s/L'_K)k'_2l'-({{{\tilde{L}}}}_s/{{{\tilde{L}}}}_K)k'_1{{{\tilde{l}}}}-(L_s/L_K)k_3l/3]$ to give rise to the relations similar to those in Eqs. (5), (6) and (7) where $k'_1l'$’s are replaced with $k'_1{{{\tilde{l}}}}$. From these relations in conjunction with the relations in Eq. (8) we can similarly calculate $k_i$ and $k'_i$ with $i=1,2,3$ in terms of $\varphi _i$ and $\varphi '_i$ (see the Supplementary Information).

In order to induce current flowing between the branches across $\varphi '_1$, we initially apply the flux $\Phi _{\mathrm{ext,1}}$ so that $f_1=\Phi _{\mathrm{ext,1}}/\Phi _0=f$, but $f_2=f_3=0$. We then can easily check that the following effective potential satisfies the equation of motion in Eqs. (11) and (13),

18 $$\begin{aligned} U_{\mathrm{eff}}(\{\varphi _i,\varphi '_i\})= & {} \frac{3\Phi ^2_0}{4{{{\tilde{L}}}}_{\mathrm{eff}}}\left( -m_2+m_3+\frac{\varphi _2 +\varphi '_2}{2\pi }-\frac{\varphi _3+\varphi '_3}{2\pi }\right) ^2 +\frac{1}{2}\left( \frac{\Phi ^2_0}{2L'_{\mathrm{eff}}} +\frac{\Phi ^2_0}{L_{\mathrm{eff}}}\right) \left( n+f-\frac{\varphi _1+\varphi _2 +\varphi _3}{2\pi }\right) ^2\nonumber \\&-\frac{3\Phi ^2_0}{2L'_{\mathrm{eff}}}\left( m_1+f-\frac{\varphi _1 +\varphi '_1}{2\pi }\right) \left( n+f-\frac{\varphi _1+\varphi _2 +\varphi _3}{2\pi }\right) +\frac{9\Phi ^2_0}{4L'_{\mathrm{eff}}} \left( m_1+f-\frac{\varphi _1+\varphi '_1}{2\pi }\right) ^2\\&-\sum ^3_{i=1}(E_{J}\cos \varphi _i+E'_{J}\cos \varphi '_i),\nonumber \end{aligned}$$

where ${{{\tilde{L}}}}_{\mathrm{eff}}\equiv L_K+L_s+3(L'_K+L'_s)+6({{{\tilde{L}}}}_K+{{{\tilde{L}}}}_s)$ is the effective inductance of the central branch. By manipulating the third term in Eq. (18) (see the Supplementary Information) we can obtain the effective potential of the system in Fig. 1b as

19 $$\begin{aligned} U_{\mathrm{eff}}(\{\varphi _i,\varphi '_i\})= & {} \frac{3\Phi ^2_0}{2L'_{\mathrm{eff}}} \left( m_1+f-\frac{\varphi _1+\varphi '_1}{2\pi }\right) ^2 +\frac{3}{2}\left( \frac{\Phi ^2_0}{2L'_{\mathrm{eff}}}+\frac{\Phi ^2_0}{2{{{\tilde{L}}}}_{\mathrm{eff}}}\right) \left[ \left( m_2-\frac{\varphi _2+\varphi '_2}{2\pi }\right) ^2 +\left( m_3-\frac{\varphi _3+\varphi '_3}{2\pi }\right) ^2 \right] \\&+\left( \frac{3\Phi ^2_0}{2L'_{\mathrm{eff}}}-\frac{3\Phi ^2_0}{2{{{\tilde{L}}}}_{\mathrm{eff}}}\right) \left( m_2-\frac{\varphi _2+\varphi '_2}{2\pi }\right) \left( m_3-\frac{\varphi _3+\varphi '_3}{2\pi }\right) \nonumber \\&+\left( \frac{\Phi ^2_0}{2L_{\mathrm{eff}}}-\frac{\Phi ^2_0}{2L'_{\mathrm{eff}}} \right) \left( n+f-\frac{\varphi _1+\varphi _2+\varphi _3}{2\pi }\right) ^2\nonumber \\&-\sum _i(E_{Ji}\cos \varphi _i+E'_{Ji}\cos \varphi '_i).\nonumber \end{aligned}$$

If we consider that the inductances of left, right and central branches are all equal, ${{{\tilde{l}}}}=l'$, ${\tilde{L}}_s=L'_s$, ${{{\tilde{L}}}}_K=L'_K$, and thus ${{{\tilde{L}}}}_\text{eff}=L'_{\text{eff}}$, the effective potential $U_\text{eff}(\{\varphi _i,\varphi '_i\})$ in Eq. (19) can be reduced to $U_{\mathrm{eff}}(\{\varphi _i,\varphi '_i\})$ in Eq. (12) for the system in Fig. 1a with $f_1=f$ and $f_2=f_3=0$. Figure 2 shows the effective potential for the design in Fig. 1b, which is qualitatively similar to that for the model in Fig. 1a.

We introduce a coordinate transformation such as $\varphi _p=(\varphi _2+\varphi _3)/2, \varphi _m=(\varphi _2-\varphi _3)/2, \varphi '_p=(\varphi '_2+\varphi '_3)/2,$ and $\varphi '_m=(\varphi '_2-\varphi '_3)/2$. The effective potential in Eq. (19), then, can be expressed as

20 $$\begin{aligned} U_{\mathrm{eff}}(\varphi _p,\varphi _m,\varphi '_p,\varphi '_m,\varphi _1)= & {} \frac{3\Phi ^2_0}{2L'_{\mathrm{eff}}}\left( m_1-n'+f-\frac{\varphi _1 -2\varphi '_p}{2\pi }\right) ^2 +\left( \frac{\Phi ^2_0}{2L_{\mathrm{eff}}}-\frac{\Phi ^2_0}{2L'_{\mathrm{eff}}} \right) \left( n+f-\frac{\varphi _1+2\varphi _p}{2\pi }\right) ^2\nonumber \\&+\frac{3}{2}\left( \frac{\Phi ^2_0}{2L'_{\mathrm{eff}}}+\frac{\Phi ^2_0}{2{{{\tilde{L}}}}_{\mathrm{eff}}}\right) \left[ \left( m_2-\frac{\varphi _p+\varphi _m+\varphi '_p+\varphi '_m}{2\pi } \right) ^2\right. \nonumber \\&\left. +\left( m_3-\frac{\varphi _p-\varphi _m+\varphi '_p-\varphi '_m}{2\pi } \right) ^2 \right] \nonumber \\&+\frac{3}{2}\left( \frac{\Phi ^2_0}{2L'_{\mathrm{eff}}}-\frac{\Phi ^2_0}{2{{{\tilde{L}}}}_{\mathrm{eff}}}\right) \left( m_2-\frac{\varphi _p+\varphi _m+\varphi '_p+\varphi '_m}{2\pi }\right) \left( m_3-\frac{\varphi _p-\varphi _m+\varphi '_p-\varphi '_m}{2\pi }\right) \\&-E_{J}\cos \varphi _1-2E_{J}\cos \varphi _p\cos \varphi _m -E'_{J}\cos 2\varphi '_p-2E'_{J}\cos \varphi '_p\cos \varphi '_m, \nonumber \end{aligned}$$

where we use $\varphi '_1=2\pi n'-(\varphi '_2+\varphi '_3)=2\pi n'-2\varphi '_p$. Figure 2a shows the effective potential $U_{\mathrm{eff}}$ as a function of $(\varphi _p,\varphi _m)$ for $m_1=m_2=m_3=n=n'=0$, which is minimized with respect to $\varphi '_p, \varphi '_m$ and $\varphi _1$. If the value of the external flux $f=0.5$, two degenerate current states, clockwise and counterclockwise, are superposed so that we cannot determine the direction of current. We thus set the value of the external flux $f=0.42$ to obtain a stable minimum. The effective potential $U_{\mathrm{eff}}(\varphi _p,\varphi _m)$ along the dotted line in Fig. 2a is shown in Fig. 2b, where $U_{\mathrm{eff}}(\varphi _p,\varphi _m)$ has a minimum at $\varphi _p/2\pi \approx 0.124$. Figure 2c shows the profile of effective potential $U_{\mathrm{eff}}(\varphi _p,\varphi _m)$ as a function of $\varphi _m$ for $\varphi _p/2\pi \approx 0.124$. Here the effective potential has the minimum at $\varphi _m=0, i. e., \varphi _2=\varphi _3$. Figure 2d show that $\varphi '_m=0, i. e., \varphi '_2=\varphi '_3$ at the minimum of the effective potential $U_{\mathrm{eff}}(\varphi _p,\varphi _m)$. From Eqs. (4) and (9) we can see that $k_2=k_3$ and thus $I_2=I_3$ and from Eq. (10) $k'_1=0$, and thus $I'_1=0$, which is consistent with the current conservations, $I_3-I_2=I'_1=0$, in Eq. (8). Hence, in Fig. 1b we can determine the direction of current such as $I'_3=-I'_2\ne 0$ , and $I'_1=0$. If we consider the case that $f_3=f, f_1=f_2=0$ or $f_2=f, f_1=f_3=0$, the currents become $I'_2=-I'_1\ne 0$, $I'_3=0$ or $I'_3=-I'_1\ne 0$, $I'_2=0$, respectively. Hence we can selectively determine the direction of currents flowing through a trijunction by threading a magnetic flux into a specific loop in the design of Fig. 1b, which can realize the circulator function in a scalable design.

### Braiding of Majorana zero modes

We can use the circulator function for the braiding of Majorana zero modes (MZM) for topological quantum computing. As shown in Fig. 4a we introduce three pairs of MZMs in the semiconducting nanowire with p-wave-like superconductivity induced from s-wave superconducting branch via proximity effect. For the quantum computing the scheme for quantum gate operation should be provided. Hence we consider the system of Fig. 1b because for the system of Fig. 1a the MZMs are inside of the loop so that the MZMs cannot interact with MZMs outside^23^.

**Figure 3:**
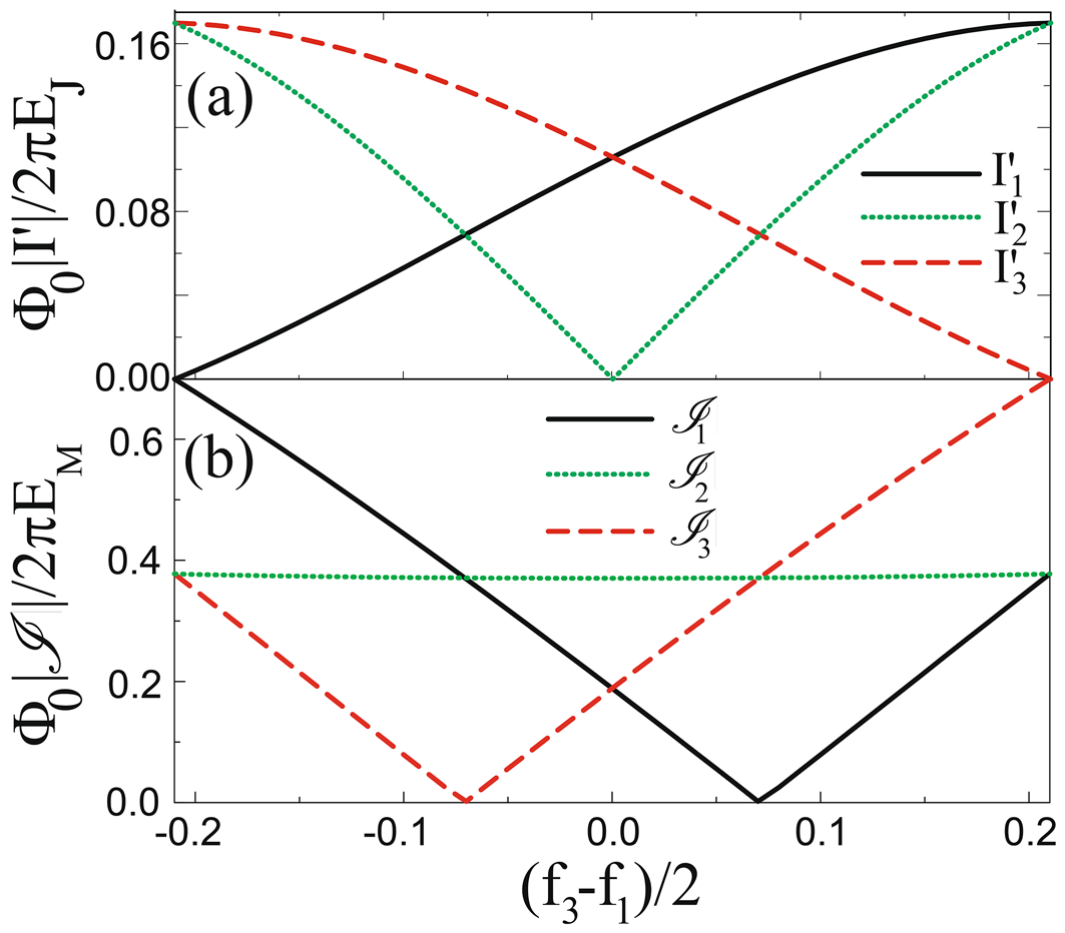
(**a**) Currents $I'_i$ in branches as a function of $(f_3-f_1)/2$. When $f_1$ starts from $f_1=0.42$ with $f_2=f_3=0$, the currents $|I'_1|=|I'_3|\ne 0$ with $|I'_1|$=0. As $f_3$ increases while $f_1$ decreases to zero, the current flow changes so that $|I'_1|=|I'_2|\ne 0$ with $|I'_3|=0$. (**b**) Currents ${{{\mathscr {I}}}}_i$ carried through MZMs across trijunction. For $f_1=0.42$ and $f_2=f_3=0$ the current ${{{\mathscr {I}}}}_1$ has larger amplitude than $|{{{\mathscr {I}}}}_2|=|{{{\mathscr {I}}}}_3|$, but for $f_3=0.42$ and $f_1=f_2=0$, $|{{{\mathscr {I}}}}_3|$ becomes larger, so the asymmetry is changed.

**Figure 4:**
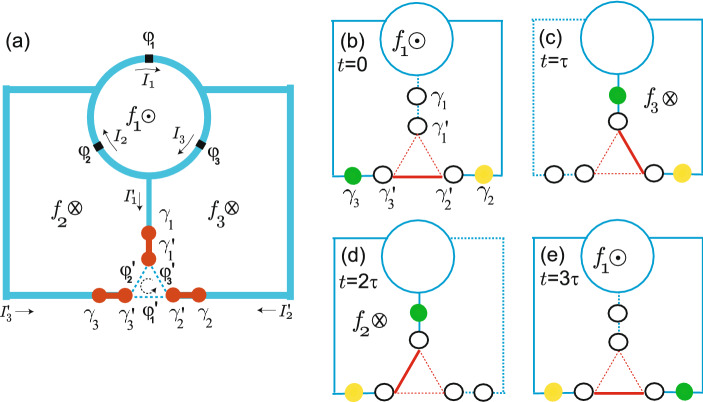
(**a**) Three MZM (red circle) pairs are introduced at the end of branches where three MZMs, $\gamma '_i$, are coupled through a Josephson trijunction. Braiding sequence of system in (**a**): by applying adiabatically the fluxes (**b**) $f_1,$ (**c**) $f_3$, (**d**) $f_2$, and finally (**e**) $f_1$ again, the green and yellow MZMs are exchanged with each other to complete a non-Abelian braiding procedure. In the branches represented as dotted line there is no current flowing. In trijunction thick red line corresponds to a large current amplitude of ${{{\mathscr {I}}}}_i$.

**Figure 5:**
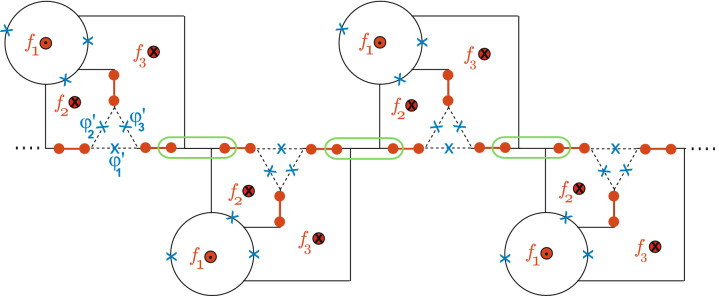
A scalable design for a superconducting circuit with MZMs. Two MZMs in each circuit of Fig. 4a can be coupled in the green box to form a one-dimensional lattice structure.

In Fig. 3a we show the currents $I'_1=I_3-I_2$, $I'_2=I_1-I_3,$ and $I'_3=I_2-I_1$ of the system in Fig. 1b as a function of $f_1-f_2$. If $f_1=f=0.42$ with $f_2=f_3=0$, the current direction is determined such that $I'_1=0$, but $I'_2=I'_3\ne 0$. In this case the current flows between the branch with $\gamma _2$ and the branch with $\gamma _3$. This is the initial state of the system shown in Fig. 4b, where the three MZMs, $\gamma '_1,\gamma '_2$ and $\gamma '_3$, are tunnel-coupled with each other through the Hamiltonian^22,23^

21 $$\begin{aligned} H_T=iE_M\left( \gamma '_1\gamma '_2\cos \frac{\varphi '_3}{2} +\gamma '_2\gamma '_3\cos \frac{\varphi '_1}{2} +\gamma '_3\gamma '_1\cos \frac{\varphi '_2}{2}\right) +i\alpha \sum ^3_{i=1}\gamma _i\gamma '_i \end{aligned}$$

with Majorana Josephson energy $E_M$ and coupling energy $\alpha$. Then the current carried through MZMs across trijunction is given by

22 $$\begin{aligned} {{{\mathscr {I}}}}_{i}=\frac{2e}{\hbar }\frac{\partial }{\partial \varphi '_i}H_T=-\frac{2\pi E_M}{\Phi _0}i\gamma '_{i+1}\gamma '_{i+2}\sin \frac{\varphi '_i}{2} \end{aligned}$$

with a $4 \pi$-periodic behavior^33^. Actually we have $\varphi '_1/2\pi \approx 0.246$ and $\varphi '_2/2\pi =\varphi '_3/2\pi \approx -0.123$ at the minimum of the effective potential $U_{\mathrm{eff}}(\varphi _p,\varphi _m)$ in Fig. 2a. Then the current ${{{\mathscr {I}}}}_1$ has a larger amplitude than ${{{\mathscr {I}}}}_2={{{\mathscr {I}}}}_3$ as shown in Fig. 3b, which is denoted as a solid (dotted) line for ${{{\mathscr {I}}}}_1 ({{{\mathscr {I}}}}_2$ and ${{{\mathscr {I}}}}_3)$ in the trijunction of Fig. 4b. As shown in Eq. (22) the current mediated by MZMs $\mathcal{I}_i\propto \sin \varphi '_i/2$, while the Cooper pair current ${{{\tilde{I}}}}_i\propto \sin \varphi '_i$. If we consider a simplified model such that the Josephson junctions in the three-junction loop in Fig. 1a are removed as in the previous study^23^, the boundary condition becomes approximately $\varphi '_i-2\pi f_i\approx 0$. Here, even if we set $f_i= 0.5$ and thus $\varphi '_i\approx \pi$, we cannot switch off the current mediated by MZMs as ${{{\mathscr {I}}}}_i \ne 0$ while ${{{\tilde{I}}}}_i\approx 0$. Hence, instead of switching off ${{{\mathscr {I}}}}_i$ we change the current direction by using circulating function to perform the braiding operation.

In general, for $f_i=0.42$ with $f_{i\pm 1}=0$ we have $\varphi '_i/2\pi \approx 0.246$ and $\varphi '_{i\pm 1}/2\pi \approx -0.123$. The different phases are due to the current direction, resulting in the asymmetry in the amplitude of ${{{\mathscr {I}}}}_i$ at the trijunction. In next stage we adiabatically apply the flux $f_3$, while decreasing $f_1$ (See Eq. (S27) of Supplementary Information for general $f_i$). In Fig. 3a, then, $|I'_1|$ increases while $|I'_3|$ decreases. In the meanwhile, $|I'_2|$ decreases to zero and then grows up to the maximum value. Finally for $f_3=0.42$ with $f_1=f_2=0$, we have $I'_3=0$, but $I'_1=I'_2\ne 0$. Hence the current direction is changed: the current ${{{\mathscr {I}}}}_i$ flows between the branch with $\gamma _1$ and the branch with $\gamma _2$ but there is no current in the branch with $\gamma _3$ as shown in Fig. 4c, and meanwhile the green MZM loses its weight in $\gamma _3$ and gains weight in $\gamma _1$. Here the current ${{{\mathscr {I}}}}_3$ has a larger amplitude than ${{{\mathscr {I}}}}_1={{{\mathscr {I}}}}_2$, and thus the asymmetry in the amplitude of ${{{\mathscr {I}}}}_i$ is changed. In this way, between $t=\tau$ and $t=2\tau$, the yellow MZM loses its weight in $\gamma _2$ and gains weight in $\gamma _3$ as shown in Fig. 4d. At the last stage the green MZM loses its weight in $\gamma _1$ and gains weight in $\gamma _2$. As a result, the green and yellow MZMs are exchanged with each other as shown in Fig. 4e, completing the braiding operation.

In Fig. 5 we show an architecture for a scalable design for a superconducting circuit with MZMs. Two MZMs belong to different trijunctions (the green box in Fig. 5) can be coupled or fused to perform quantum gate operations and quantum measurements. For the green box operation, for example, we can introduce a gate voltage applied to the sector between two MZMs to control the chemical potential of the nanowire^34^. Though the system in Fig. 5 is one-dimensional, we can extend it to two-dimensional lattice straightforwardly.

## Discussion

In conclusion, we proposed a scheme for the circulator function in a superconducting circuit consisting of three small loops and branches which meet at a trijunction. Usually the effective potential in the Hamiltonian for superconducting circuit is phenomenologically obtained. However in this study we obtained the boundary conditions from the fundamental fluxoid quantization condition for the superconducting loop to derive the effective potential of the system analytically, which is required for accurate and systematic study for the quantum information processing applications. We expect that this kind of study can be applied to other systems.

At the minimum of the effective potential we can see that two branches carry current while the other does not. By applying a magnetic flux into one of the loops we can determine which branches among three carry the current, achieving the circulator function. For the NISQ computing we need to perform the circulator function in a scalable design. We thus introduced an improved model where the trijunction is extracted out from the outmost loop to interact with other external branches. For the improved design we obtained the ground state of the system from the effective potential, and showed that it can perform the circulator function in the trijunction loop.

Instead of switching off the current mediated by MZMs in the previous study, in this study we selectively choose the current directions to give rise to MZM braiding. We thus use the circulator function to achieve a non-Abelian braiding operation by introducing three pairs of MZMs in the branches that meet at a trijunction in the improved model where MZMs are introduced outside of the loop. The circulator function determines the phases of the trijunction and thus the coupling between the MZMs. Initially we apply a magnetic flux into one of the three loops to selectively couple two pairs of MZMs. By applying adiabatically a flux into another loop while decreasing the previous flux we are able to gain the weight of MZM while losing in the previous branch. Consecutive executions in this way can perform the braiding operation between two MZMs. This scheme could be extended to a scalable design to implement braiding operations in one- or two-dimensional circuits.

## Supplementary information

Supplementary material 1
