# Supplementary material for: Circulator function in a Josephson junction circuit and braiding of Majorana zero modes
Source: Sci Rep. 2021 Jan 19;11:1826. doi: 10.1038/s41598-021-81503-1 (PMC7815894; doi:10.1038/s41598-021-81503-1)
Supplement: Supplementary file 1 — Supplementary material 1 [file 41598_2021_81503_MOESM1_ESM.pdf]

Supplementary Information for "Circulator function in a Josephson junction circuit and braiding of Majorana zero modes"

Mun Dae Kim

College of Liberal Arts, Hongik University, Sejong 30016, Korea

S1. EFFECTIVE POTENTIAL OF THE IMPROVED CIRCUIT

The design of Fig. 1 in the main manuscript can be simplified as Fig. S1. In the figures we denote the currents I_i and I'_i in the loop whose direction is opposite to the Cooper pair wave vector k_i and k'_i, respectively. In this Supplementary Information we consider the more general case of Fig. S1(b). Here, we consider that $f_1 = f$ and $f_2 = f_3 = 0$. The boundary conditions for the scheme in Fig. S1(b) including the phase differences across the Josephson junctions are represented as,

$$k'_2 l' - k'_3 l' + k_1 \frac{l}{3} + \varphi_1 + \varphi'_1 = 2\pi(m_1 + f + f_{\text{ind},1}), \tag{S1}$$

$$-k'_3 l' + k'_1 \tilde{l} - k_2 \frac{l}{3} - \varphi_2 - \varphi'_2 = 2\pi(-m_2 + f_{\text{ind},2}), \tag{S2}$$

$$k'_2 l' - k'_1 \tilde{l} - k_3 \frac{l}{3} - \varphi_3 - \varphi'_3 = 2\pi(-m_3 + f_{\text{ind},3}), \tag{S3}$$

with integers m_i.

Equation (S1) describes the boundary condition for the outmost loop containing the Josephson junctions with phase differences φ_1 and φ'_1, and Eqs. (S2) and (S3) the left and right loop in Fig. S1(b). The induced flux, $f_{\text{ind,i}} = \Phi_{\text{ind,i}}/\Phi_0$, can be written as

$$f_{\text{ind},1} = (1/\Phi_0)(I_1 + I_2 + I_3)L_s/3, \tag{S4}$$

$$f_{\text{ind},2} = (1/\Phi_0)(-L'_s I'_3 + \tilde{L}_s I'_1 - L_s I_2/3), \tag{S5}$$

$$f_{\text{ind},3} = (1/\Phi_0)(L'_s I'_2 - \tilde{L}_s I'_1 - L_s I_3/3), \tag{S6}$$

where the Cooper pair current I is given by

$$I_i = -(n_c A q_c/m_c)\hbar k_i. \tag{S7}$$

With the kinetic inductances of side branches, central branch, and the three-Josephson junction loop being $L'_K = m_c l'/A n_c q_c^2, \tilde{L}_K = m_c \tilde{l}/A n_c q_c^2$ and $L_K = m_c l/A n_c q_c^2$, respectively, the induced fluxes become

$$f_{\text{ind},1} = -\frac{1}{2\pi}[(L'_s/L'_K)(k'_2 - k'_3)l' + (L_s/L_K)k_1 l/3], \tag{S8}$$

$$f_{\text{ind},2} = -\frac{1}{2\pi}[-(L'_s/L'_K)k'_3 l' + (\tilde{L}_s/\tilde{L}_K)k'_1\tilde{l} - (L_s/L_K)k_2 l/3], \tag{S9}$$

$$f_{\text{ind},3} = -\frac{1}{2\pi}[(L'_s/L'_K)k'_2 l' - (\tilde{L}_s/\tilde{L}_K)k'_1\tilde{l} - (L_s/L_K)k_3 l/3]. \tag{S10}$$

Then the boundary conditions are represented as

$$\left(1+\frac{L_s}{L_K}\right)k_1\frac{l}{3} + \left(1+\frac{L'_s}{L'_K}\right)k'_2 l' - \left(1+\frac{L'_s}{L'_K}\right)k'_3 l' = 2\pi\left(m_1 + f - \frac{\varphi_1+\varphi'_1}{2\pi}\right) \tag{S11}$$

$$\left(1+\frac{L_s}{L_K}\right)k_2\frac{l}{3} + \left(1+\frac{L'_s}{L'_K}\right)k'_3 l' - \left(1+\frac{\tilde{L}_s}{\tilde{L}_K}\right)k'_1 \tilde{l} = 2\pi\left(m_2 - \frac{\varphi_2+\varphi'_2}{2\pi}\right) \tag{S12}$$

$$\left(1+\frac{L_s}{L_K}\right)k_3\frac{l}{3} + \left(1+\frac{\tilde{L}_s}{\tilde{L}_K}\right)k'_1 \tilde{l} - \left(1+\frac{L'_s}{L'_K}\right)k'_2 l' = 2\pi\left(m_3 - \frac{\varphi_3+\varphi'_3}{2\pi}\right) \tag{S13}$$

with $\tilde{L}_s$ and L'_s being the self inductance of the central and the side branch, respectively.

The current conservation conditions, $I_1 = I_3 + I'_2, I_2 = I_1 + I'_3$, and $I_3 = I_2 + I'_1$, at the nodes of three-Josephson junction loop give rise to the relations,

$$k_1 = k_3 + k'_2, k_2 = k_1 + k'_3, k_3 = k_2 + k'_1. \tag{S14}$$

From Eqs. (S11), (S12), (S13), and (S14) we can obtain

$$k_1 = \frac{2\pi}{l}\frac{3L_K}{L'_{\text{eff}}}\left(m_1 + f - \frac{\varphi_1+\varphi'_1}{2\pi}\right) + \frac{2\pi}{l}\left(\frac{L_K}{L_{\text{eff}}} - \frac{L_K}{L'_{\text{eff}}}\right)\left(n + f - \frac{\varphi_1+\varphi_2+\varphi_3}{2\pi}\right), \tag{S15}$$

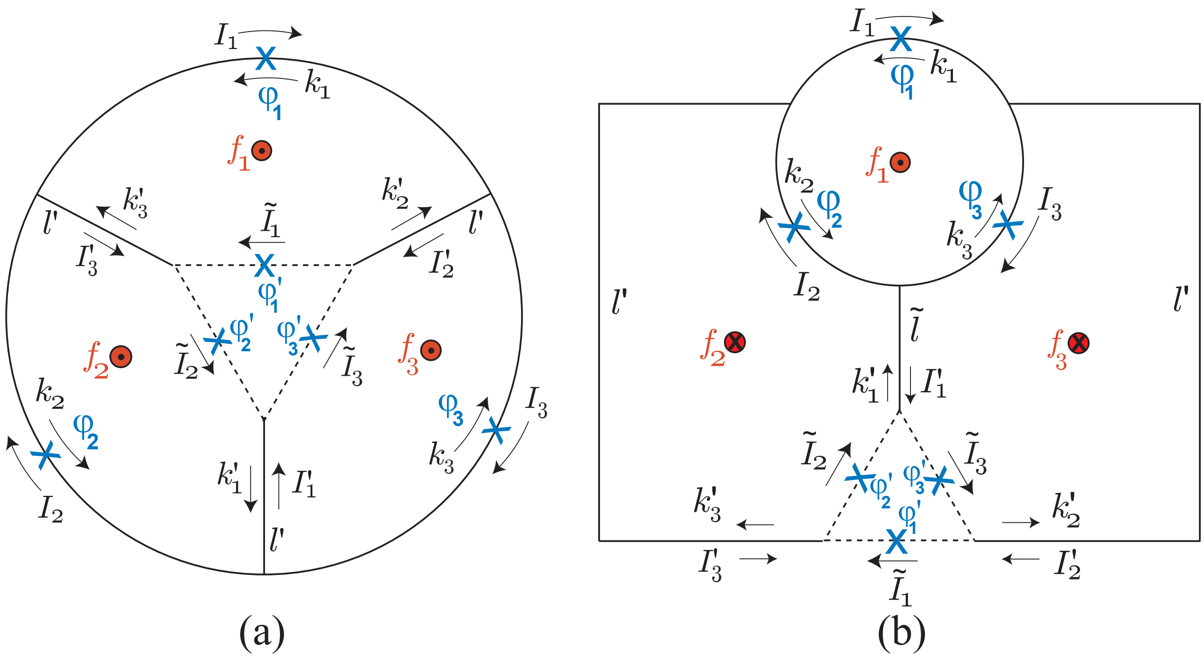

FIG. S1: Simplified picture of Fig. 1 in the main manuscript.

$$
\begin{aligned}
k_2 &= -\frac{\pi}{l}\frac{3L_K}{L'_{\text{eff}}}\left(m_1+f-\frac{\varphi_1+\varphi'_1}{2\pi}\right)-\frac{\pi}{l}\frac{3L_K}{\tilde{L}_{\text{eff}}}\left(m+\frac{\varphi_2+\varphi'_2}{2\pi}-\frac{\varphi_3+\varphi'_3}{2\pi}\right) \\
&+ \frac{2\pi}{l}\left(\frac{L_K}{L_{\text{eff}}}+\frac{L_K}{2L'_{\text{eff}}}\right)\left(n+f-\frac{\varphi_1+\varphi_2+\varphi_3}{2\pi}\right), \qquad \text{(S16)} \\
k_3 &= -\frac{\pi}{l}\frac{3L_K}{L'_{\text{eff}}}\left(m_1+f-\frac{\varphi_1+\varphi'_1}{2\pi}\right)+\frac{\pi}{l}\frac{3L_K}{\tilde{L}_{\text{eff}}}\left(m+\frac{\varphi_2+\varphi'_2}{2\pi}-\frac{\varphi_3+\varphi'_3}{2\pi}\right) \\
&+ \frac{2\pi}{l}\left(\frac{L_K}{L_{\text{eff}}}+\frac{L_K}{2L'_{\text{eff}}}\right)\left(n+f-\frac{\varphi_1+\varphi_2+\varphi_3}{2\pi}\right), \qquad \text{(S17)} \\
k'_1 &= \frac{2\pi}{l}\frac{3L_K}{\tilde{L}_{\text{eff}}}\left(m+\frac{\varphi_2+\varphi'_2}{2\pi}-\frac{\varphi_3+\varphi'_3}{2\pi}\right), \qquad \text{(S18)} \\
k'_2 &= -\frac{\pi}{l}\frac{3L_K}{\tilde{L}_{\text{eff}}}\left(m+\frac{\varphi_2+\varphi'_2}{2\pi}-\frac{\varphi_3+\varphi'_3}{2\pi}\right) \\
&- \frac{\pi}{l}\frac{9L_K}{L'_{\text{eff}}}\left[m_1-f+\frac{\varphi_1+\varphi'_1}{2\pi}+\frac{1}{3}\left(n+f-\frac{\varphi_1+\varphi_2+\varphi_3}{2\pi}\right)\right], \qquad \text{(S19)} \\
k'_3 &= -\frac{\pi}{l}\frac{3L_K}{\tilde{L}_{\text{eff}}}\left(m+\frac{\varphi_2+\varphi'_2}{2\pi}-\frac{\varphi_3+\varphi'_3}{2\pi}\right) \\
&+ \frac{\pi}{l}\frac{9L_K}{L'_{\text{eff}}}\left[m_1-f+\frac{\varphi_1+\varphi'_1}{2\pi}+\frac{1}{3}\left(n+f-\frac{\varphi_1+\varphi_2+\varphi_3}{2\pi}\right)\right], \qquad \text{(S20)}
\end{aligned}
$$

where $L_{\text{eff}} = L_K + L_s$, $L'_{\text{eff}} = L_K + L_s + 9(L'_K + L'_s)$, and $\tilde{L}_{\text{eff}} \equiv L_K + L_s + 3(L'_K + L'_s) + 6(\tilde{L}_K + \tilde{L}_s)$ are the effective inductances of three-Josephson junction loop, side branches, and central branch, respectively.

By using the quantum Kirchhoff relation the equation of motion can be represented as

$$
\begin{aligned}
\frac{\Phi_0^2}{2\pi L_K}\frac{l}{2\pi}k_i - E_J\sin\phi_i &= -\frac{\partial U_{\text{eff}}}{\partial\phi_i}, \qquad \text{(S21)} \\
-\frac{\Phi_0^2}{2\pi L_K}\frac{l}{2\pi}k'_i &= \frac{\partial U_{\text{eff}}}{\partial\varphi'_{i+2}} - \frac{\partial U_{\text{eff}}}{\partial\varphi'_{i+1}} - E'_J\sin\varphi'_{i+2} + E'_J\sin\varphi'_{i+1}. \qquad \text{(S22)}
\end{aligned}
$$

We then can obtain the effective potential satisfying Eqs. (S21) and (S22) as follows,

$$
\begin{aligned}
U_{\text{eff}}(\{\varphi_i,\varphi'_i\}) &= \frac{3\Phi_0^2}{4\tilde{L}_{\text{eff}}}\left(-m_2+m_3+\frac{\varphi_2+\varphi'_2}{2\pi}-\frac{\varphi_3+\varphi'_3}{2\pi}\right)^2+\frac{1}{2}\left(\frac{\Phi_0^2}{2L'_{\text{eff}}}+\frac{\Phi_0^2}{L_{\text{eff}}}\right)\left(n+f-\frac{\varphi_1+\varphi_2+\varphi_3}{2\pi}\right)^2 \\
&- \frac{3\Phi_0^2}{2L'_{\text{eff}}}\left(m_1+f-\frac{\varphi_1+\varphi'_1}{2\pi}\right)\left(n+f-\frac{\varphi_1+\varphi_2+\varphi_3}{2\pi}\right)+\frac{9\Phi_0^2}{4L'_{\text{eff}}}\left(m_1+f-\frac{\varphi_1+\varphi'_1}{2\pi}\right)^2 \\
&- \sum_i (E_{Ji}\cos\varphi_i + E'_{Ji}\cos\varphi'_i). \qquad \text{(S23)}
\end{aligned}
$$

The third term of Eq. (S23) can be rewritten as

$$
\begin{aligned}
&\frac{3\Phi_0^2}{4L'_{\text{eff}}}\left[\left(n+f-\frac{\varphi_1+\varphi_2+\varphi_3}{2\pi}\right)-\left(m_1+f-\frac{\varphi_1+\varphi'_1}{2\pi}\right)\right]^2 \\
&-\frac{3\Phi_0^2}{4L'_{\text{eff}}}\left(n+f-\frac{\varphi_1+\varphi_2+\varphi_3}{2\pi}\right)^2-\frac{3\Phi_0^2}{4L'_{\text{eff}}}\left(m_1+f-\frac{\varphi_1+\varphi'_1}{2\pi}\right)^2, \qquad \text{(S24)}
\end{aligned}
$$

where by using $\varphi_1' = 2\pi m' - \varphi_2' - \varphi_3'$ and choosing appropriate m' the first term of Eq. (S24) can be represented as

$$\frac{3\Phi_0^2}{4L'_{\rm eff}}\left(m_2 - \frac{\varphi_2+\varphi_2'}{2\pi} + m_3 - \frac{\varphi_3+\varphi_3'}{2\pi}\right)^2. \tag{S25}$$

As a result, the effective potential $U_{\rm eff}(\{\varphi_i, \varphi_i'\})$ in Eq. (S23) is reexpressed as follows,

$$\begin{aligned}U_{\rm eff}(\{\varphi_i,\varphi_i'\})=&\frac{3\Phi_0^2}{2L'_{\rm eff}}\left(m_1+f-\frac{\varphi_1+\varphi_1'}{2\pi}\right)^2+\frac{3}{2}\left(\frac{\Phi_0^2}{2L'_{\rm eff}}+\frac{\Phi_0^2}{2\tilde{L}_{\rm eff}}\right)\left[\left(m_2-\frac{\varphi_2+\varphi_2'}{2\pi}\right)^2+\left(m_3-\frac{\varphi_3+\varphi_3'}{2\pi}\right)^2\right]\\ &+\left(\frac{\Phi_0^2}{2L_{\rm eff}}-\frac{\Phi_0^2}{2L'_{\rm eff}}\right)\left(n+f-\frac{\varphi_1+\varphi_2+\varphi_3}{2\pi}\right)^2-\sum_i(E_{Ji}\cos\varphi_i+E'_{Ji}\cos\varphi_i').\\ &+\left(\frac{3\Phi_0^2}{2L'_{\rm eff}}-\frac{3\Phi_0^2}{2\tilde{L}_{\rm eff}}\right)\left(m_2-\frac{\varphi_2+\varphi_2'}{2\pi}\right)\left(m_3-\frac{\varphi_3+\varphi_3'}{2\pi}\right).\end{aligned} \tag{S26}$$

If we consider the general case that $f_1 \neq 0, f_2 \neq 0$ and $f_3 \neq 0$ with $f_x = f_1 + f_2 + f_3$, the effective potential can be obtained straightforwardly as

$$\begin{aligned}U_{\rm eff}(\{\varphi_i,\varphi_i'\})=&\frac{3\Phi_0^2}{2L'_{\rm eff}}\left(m_1+f_1-\frac{\varphi_1+\varphi_1'}{2\pi}\right)^2\\ &+\frac{3}{2}\left(\frac{\Phi_0^2}{2L'_{\rm eff}}+\frac{\Phi_0^2}{2\tilde{L}_{\rm eff}}\right)\left[\left(m_2+f_2-\frac{\varphi_2+\varphi_2'}{2\pi}\right)^2+\left(m_3+f_3-\frac{\varphi_3+\varphi_3'}{2\pi}\right)^2\right]\\ &+\left(\frac{\Phi_0^2}{2L_{\rm eff}}-\frac{\Phi_0^2}{2L'_{\rm eff}}\right)\left(n+f_x-\frac{\varphi_1+\varphi_2+\varphi_3}{2\pi}\right)^2-\sum_i(E_{Ji}\cos\varphi_i+E'_{Ji}\cos\varphi_i').\\ &+\left(\frac{3\Phi_0^2}{2L'_{\rm eff}}-\frac{3\Phi_0^2}{2\tilde{L}_{\rm eff}}\right)\left(m_2+f_2-\frac{\varphi_2+\varphi_2'}{2\pi}\right)\left(m_3+f_3-\frac{\varphi_3+\varphi_3'}{2\pi}\right).\end{aligned} \tag{S27}$$
